# Between Conspiracy Beliefs, Ingroup Bias, and System Justification: How People Use Defense Strategies to Cope With the Threat of COVID-19

**DOI:** 10.3389/fpsyg.2020.578586

**Published:** 2020-09-30

**Authors:** Chiara A. Jutzi, Robin Willardt, Petra C. Schmid, Eva Jonas

**Affiliations:** ^1^Department of Psychology, University of Salzburg, Salzburg, Austria; ^2^Department of Management, Technology, and Economics, ETH Zürich, Zurich, Switzerland

**Keywords:** COVID-19, threat, defense strategies, conspiracy, system justification, worldview defense, BIS, BAS

## Abstract

The current situation around coronavirus disease 2019 (COVID-19) portrays a threat to us in several ways: It imposes uncertainty and a lack of control and reminds us of our own mortality. People around the world have reacted to these threats in seemingly unrelated ways: From stockpiling yeast and toilet paper to favoring nationalist ideas or endorsing conspiratorial beliefs. According to the *General Process Model of Threat and Defense*, the confrontation with a threat – a discrepant experience – makes humans react with both proximal and distal threat responses. While the proximal response manifests in behavioral inhibition that leads to heightened anxious arousal and vigilance, distal responses seek to lower behavioral inhibition and the associated state of anxiety and vigilance through engaging in distal defenses. In the present research, we propose that the reactions to COVID-19 may represent distal defense strategies to the pandemic and, therefore, can be explained and forecasted by the model. Thus, we hypothesized increased perceived COVID-19 threat to lead to a proximal threat response in the form of heightened behavioral inhibition. This, in return, should enhance the use of distal defenses (i.e., several ingroup biases, system justification, and conspiratorial beliefs) overlapping with the reactions observed as a response to COVID-19. This hypothesized mediated effect of increased perceived COVID-19 threat on distal defenses was tested in two preregistered studies: In Study 1 (*N* = 358), results showed perceived COVID-19 threat to be related to behavioral inhibition and, in turn, to be associated with increased distal defenses (i.e., higher entitativity, control restoration motivation, passive party support). In Study 2 (*N* = 348), we manipulated COVID-19 threat salience and found results suggesting the distal defenses of ingroup entitativity, system justification, and conspiratorial beliefs to be mediated by the proximal threat response. The results of the present research hint toward a common mechanism through which the seemingly unrelated reactions to COVID-19 can be explained. The results might help to predict future behavior during the COVID-19 pandemic and to design measures to counteract the detrimental effects of the pandemic.

As of August 27th, 2020, the coronavirus has claimed the lives of more than 800,000 people, while a further 24 million have been infected with the virus (Johns Hopkins Coronavirus Resource Center, 2020). Governmental countermeasures such as curfews and border closures have a detrimental impact on the economy, bringing personal restrictions as well as uncertainty into our everyday lives. Hence, UN secretary António Guterres has called coronavirus disease 2019 (COVID-19) the biggest threat since World War II (Saxena et al., 2020). Simultaneously to the rise of this threat, we observe a variety of reactions such as the spread of and belief in conspiracies (Šrol et al., 2020) as well as nationalist (Bieber, 2020) and ingroup-focused attitudes and behaviors to the pandemic (OE3, 2020). Moreover, political leaders across numerous countries seem to celebrate an all-time high in appreciation as people turn to justify the systems they live in Ehni (2020). Partly, these reactions lead to positive consequences such as increased solidarity and cooperation within countries (Cappelen et al., 2020; United Nations Development Programme [UNDP], 2020). At the same time, they trigger dangerous developments such as resentments against fellow citizens of Asian descent (Lee, 2020; Liu, 2020). Therefore, the question of why and how COVID-19 might lead to reactions such as increased ingroup bias, system justification, and conspiracy beliefs arises.

We proposed and tested whether the reactions to COVID-19 can be explained by the motivational process outlined in the *General Process Model of Threat and Defense* (Jonas et al., 2014). The model states that the threats incorporated in the current pandemic, such as the salience of one’s own death and a lack of control, trigger the behavioral inhibition system (BIS), which is associated with increased vigilance and anxiety as a first proximal threat reaction. In order to overcome this state of inhibition, to lower anxiety, and to reestablish agency, people engage in distal defense strategies. The distal defense strategies proposed by the General Process Model of Threat and Defense (Jonas et al., 2014) can occur on a behavioral as well as on a cognitive level and do not need to tackle the threat at hand. Such distal defenses may include (but are not limited to) various worldview defenses, such as increased ingroup bias, a higher belief in conspiracies, and a greater level of justification for the political system one is living in – phenomena that we are currently observing in response to COVID-19 as well. The question thus emerges whether the model can be used to explain the origins of COVID-19 reactions.

In the present research, we hypothesized based on the General Process Model of Threat and Defense (Jonas et al., 2014) that enhanced levels of perceived COVID-19 threat are associated with a greater activation of the BIS as a proximal threat reaction. To overcome the ensuing anxiety, vigilance, and behavioral inhibition, we expected this enhanced activation of the BIS to be related to an increased use of distal defense strategies in the form of the seemingly irrational reactions outlined above.

## Literature Review

### COVID-19 – A Super Threat

We argue that COVID-19 leads to distal defenses as a response to feeling threatened. This raises the question of what exactly constitutes the threat that is imposed on us by COVID-19. Foremost, the virus threatens the lives of us all: Even though older people are especially vulnerable to a severe disease progression, young people and children can die from it (Cha, 2020; McKie, 2020) as well. This awareness of our own finiteness represents a natural death reminder (Pyszczynski et al., 2006; Burke et al., 2010), which represents one of the most often discussed threats in past literature. Due to the awareness of the physical danger of the virus, governments around the globe have reacted with measures such as curfews, contact limitations (Stanford, 2020), and closed borders (Cherelus, 2020) aimed at slowing down or even completely preventing the spread of the virus. These measures pose an additional threat to us, as they restrict our behavior and disrupt our everyday lives: Self-determined actions such as going to work, meeting friends and family, or going on holidays have been eradicated from our schedules. This lack of control has been considered a threat and was found to lead to compensatory re-affirmation in other domains of people’s personal lives (Fritsche et al., 2013). Furthermore, at this time, many questions regarding the disease remain unanswered: Will there be a second wave of infections? How long will it be until medication and a vaccination are available? Will the healthcare system and the economy break down as a result of curfews? These unanswered questions and the resulting uncertainty pose another threat to us (Van den Bos, 2009). Hence, COVID-19 can be defined as a combination of several subthreats that together create a “COVID-19 super threat” (Jutzi et al., under review).

### Reactions to COVID-19 as a Super Threat

People around the globe have reacted in manifold ways to the threats of COVID-19. Early on during the pandemic, people started to stockpile everyday goods such as toilet paper without any indication for supply shortages (Erhöhte Nachfrage - aber keine Engpässe, 2020). Often even without knowing how to use it, yeast was being purchased in large quantities (Guynn and Tyko, 2020). With occasional unavailability of the mentioned goods at supermarkets (n-tv, 2020) as well as some clogged toilets due to misusing other items as toilet paper (Siemaszko, 2020), the consequences of these instances of panic buying can generally be described as negligible.

The same cannot be stated for group-related reactions: With the spread of the virus, citizens of countries around the world have shown an increase in identification and support for their own national ingroup (Bieber, 2020). At times, this has led to unprecedented acts of collective cooperation and altruistic actions such as shopping for the elderly or sewing face masks (Newman, 2020). At the same time, nationalist tendencies (Bieber, 2020) and resentments against outgroups, especially those associated with COVID-19 (e.g., Chinese citizens, Asian-Americans) have been on the rise, too (Lee, 2020). As a consequence, increased discrimination against these groups were registered across countries (Reny and Barreto, 2020; Schild et al., 2020).

In addition to these group-related reactions, COVID-19 also led to several reactions toward people’s environment. Firstly, many citizens accepted and even endorsed the restrictions of personal rights imposed on them by their governments. The defense and justification of the political *status quo* has been described as system justification (Jost and Andrews, 2011). Increased system justification tendencies mean that introduced political measures are accepted in the public (Schulte von Drach, 2020) and can better unfold to counteract the pandemic. At the same time, certain leaders have used this heightened system justification to push personal agendas (Phillips, 2020) and abolish important aspects of the democratic system in their respective countries (Schmidt, 2020).

Secondly, an increase of belief in corona-related conspiracies can be observed since the outbreak of the pandemic (Paternoster et al., 2020). Allegations that COVID-19 is actually not more dangerous than the flu (Yan and Esparza, 2020), that the virus was created intentionally in a Chinese laboratory (Borger, 2020; Stellino, 2020), or that Bill Gates is using the virus to enforce chip implantations through forced vaccinations (Goodman and Carmichael, 2020; Weiss and Greenstreet, 2020) are being pushed by a substantial amount of people (Freeman et al., 2020). For some, these theories compromised the trust in scientific knowledge and advice from virologists. The resulting public gatherings and demonstrations against governmental measures help the virus to spread further (Cipriano, 2020; Rabin, 2020).

The described COVID-19 reactions differ regarding their nature and the severity of their consequences and bear tremendous risks but also chances on a personal as well as a societal level. This is why we focused on these reactions in the present paper. Despite the diversity of distal defense reactions, they all have in common that they do not directly reduce, let alone diminish the threats incorporated in COVID-19. Nor do they provide a remedy. Hence, the question of why and how these reactions unfold arises. By applying the General Process Model of Threat and Defense (Jonas et al., 2014), the present research aims to shed light on this question.

### The General Process Model of Threat and Defense

The General Process Model of Threat and Defense proposes a singular mechanism through which the different subthreats incorporated in COVID-19 could lead to the reactions outlined above. The model points out that despite their different nature, various threats such as the finiteness of our own life (Greenberg et al., 1986), uncertainty (McGregor, 2006; McGregor et al., 2010; Nash et al., 2011), and a lack of control (Fritsche et al., 2008) all share a common feature in that they yield “some experience of discrepancy between an expectation or desire and the current circumstances” (Jonas et al., 2014, p. 229). COVID-19 threatens one’s life, brings uncertainty and a lack of personal control, and as such, represents a combination of threat-induced discrepancies. According to the model, these discrepancies lead to similar affective, behavioral, and cognitive reactions that can be clustered into proximal and distal defense reactions.

#### Proximal and Distal Threat Reactions

According to the model, the experience of a threat-induced discrepancy starts the following cascade: Firstly, a proximal reaction in the form of an activation of the BIS (McNaughton, 1982; Gray and McNaughton, 2000) is launched that is accompanied by a state of inhibition, heightened vigilance, and anxious arousal. The General Process Model of Threat and Defense proposes that, since prolonged BIS activation is uncomfortable and causes a variety of negative consequences, it becomes necessary to downregulate BIS activation. In order to exit the state of inhibition and to overcome the threat-induced anxiety, an activation of the behavioral approach system (BAS) triggering an approach-motivated state “that mutes the BIS and relieves anxiety” (Jonas et al., 2014, p. 242) is mounted. This is caused by the use of distal defensive strategies that either offer a direct solution to the discrepancy or are merely palliative responses that direct away from the threat. When direct defenses that tackle the threat itself are unavailable – as is the case for COVID-19 for which vaccines are still to be discovered and other treatment options are sparsely available – palliative defenses remain. According to the General Process Model of Threat and Defense, all defense strategies share a common motivational feature, namely a clear commitment to either incentives, activities, goals, ideals, or groups. This commitment then triggers an approach-oriented state which is responsible for the defenses’ anxiety-lowering and BIS-muting effects. Recent results indicate that palliative defenses might even be more effective than direct ones at muting BIS and reducing anxiety (Stollberg et al., under review).

#### COVID-19 Reactions as Distal Threat Defenses

Many of the distal defense strategies against threat-induced anxiety named by the General Process Model of Threat and Defense overlap with the observed reactions to COVID-19. In the following, we will briefly describe those distal defense strategies that overlap with the reactions people have shown as a response to COVID-19. These distal defenses can be called *worldview defenses* and are defined as a “range of social psychological phenomena, such as interpersonal attraction, authoritarian behavior, nationalism, and prejudice [that] are motivated in part by the need to maintain faith in a cultural worldview that provides protection from mortality concerns” (Greenberg et al., 1994, p. 627).

#### Worldview Defenses

##### System justification

The defense of political structures or systems is a type of cultural worldview defense (Rutjens and Loseman, 2010). Past research has shown that the tendency to justify the *status quo* of one’s own political system (Jost et al., 2004) is higher under threat (Jost and Hunyady, 2005; Kay et al., 2009). This increased system justification can also be found during COVID-19: In the wake of COVID-19, drastic political measures that restricted personal rights and freedom were introduced to battle the virus. In many countries, these measures nevertheless coincided with increased approval rates for governmental institutions (Bol et al., 2020).

##### Conspiratorial thinking

Another worldview defense used under threat is conspiratorial thinking. Whitson and Galinsky (2008), for instance, found that susceptibility to false information and belief in conspiratorial ideas is greater under threat. Furthermore, others showed increased conspiratorial thinking under uncertainty (Van Prooijen and Jostmann, 2013) and a lack of control (Van Prooijen and Acker, 2015), threats that are incorporated into the General Process Model of Threat and Defense as well. As was pointed out above, belief in conspiratorial ideas saw an immense rise during the pandemic (Shahsavari et al., 2020).

##### Ingroup biases

A repeatedly observed worldview and distal defense describes a stronger identification with one’s own ingroup in the face of threat (Fritsche et al., 2008; Giannakakis and Fritsche, 2011). Following threat, anxious people tend to identify more with an ingroup that is salient in that particular moment. This phenomenon was also observed in the face of COVID-19: With statements such as “Austria has so far come through this crisis better than other countries. The reason for this is you, dear Austrians” (translated statement by Bundeskanzleramt, 2020), several states entered what might be considered a competition as to who would most efficiently find a solution to the crisis’ problems. Furthermore, patriotism and nationalist attitudes saw a rise in several countries during the COVID-19 pandemic (Bieber, 2020). Not just national but also political identities can be enhanced by threat (i.e., party affiliations, see Fritsche et al., 2008). Indirect evidence for increased political ingroup identification comes, for instance, from the United States where United States citizens identifying as Democrats blamed Trump rather than China or the World Health Organization for being responsible for the crisis induced by COVID-19 (Santucci and Cummings, 2020). One way the identification with an ingroup helps to lower BIS and anxiety is through increased control restoration motivation. This phenomenon describes the tendency to perceive a restoration of personal control through being a member of an agentic group as a distal defense against threat (Fritsche et al., 2008).

These examples of worldview defenses hint toward a substantial overlap between the distal defense strategies outlined in the General Process Model of Threat and Defense and the observed reactions to COVID-19. We therefore argue that – in line with the model – the seemingly unrelated reactions to COVID-19 can be conceptualized as distal defenses to counteract the BIS-induced anxiety and vigilance caused by the threats incorporated by the virus. The presented media reports and correlational indicators cannot be considered sufficient proof for this claim though. This as a culture of blaming political failures in a partisan way is a phenomenon that has existed before COVID-19 in the United States (Golshan, 2016). Also, other indicators hinting at the use of distal defense strategies, such as conspiracy beliefs or system justification as responses to COVID-19, must be interpreted cautiously, since the data describing the reactions to the pandemic is emerging only now. Similarly, media reports which are partially used as sources here can be biased. Hence, the outlined overlap between COVID-19 reactions on the one hand and distal threat defenses on the other does not suffice to claim that the observed reactions to COVID-19 really act as threat-induced distal defenses and therefore can be conceptualized as such. Finding empirical evidence for this claim is the purpose of the present research.

## The Present Research

To test the rise of several distal defenses to emerge as a result of the BIS- and anxiety-increasing threat properties of COVID-19, we conducted two preregistered online studies^1^. In Study 1, we hypothesized a heightened perceived threat through COVID-19 to be associated with heightened passive party support to participants’ preferred political party (H1), heightened control restoration motivation (H2), heightened ingroup bias (H3), heightened ingroup entitativity (H4), and heightened outgroup derogation (H5). We furthermore hypothesized each of the proposed associations of heightened perceived threat through COVID-19 and the defense variables outlined above to be mediated by increased behavioral inhibition (H6–H10)^2^. Hence, the preregistration of Study 1 entails five hypothesized main effects and five hypothesized mediation effects.

In Study 2, in which the threat levels of COVID-19 were experimentally manipulated, we hypothesized a high COVID-19 threat level (versus a low COVID-19 threat level) to lead to an increased use of the distal defense strategies ingroup bias (H1), ingroup entitativity (H2), system justification (H3), and conspiracy beliefs (H4). We furthermore hypothesized these effects of increased perceived corona threat on distal defense strategies to be mediated by enhanced behavioral inhibition^3^. Hence, the preregistration of Study 2 entails four hypothesized main effects and four hypothesized mediation effects.

### Study 1

The goal of Study 1 was to examine whether heightened perceived COVID-19 threat is associated with heightened activation of the BIS and, as a consequence, indirectly associated with greater use of distal defense strategies in the form of worldview defenses. Study 1 was conducted as a Qualtrics online study and study links were sent to a United States-based MTurk sample (*N*_*total*_ = 633) *via* Amazon’s Mechanical Turk.

#### Participants and Exclusions

##### Sample size

The sample size of this study was determined *via* a power analysis designed to find the number of participants needed to find the hypothesized indirect effect with a likelihood of 80%, setting alpha error probability to α = 0.05. The power analysis was conducted with Kenny (2017) MedPower application. We assumed an effect size of *r* = 0.15 for the associations between COVID-19 threat levels and behavioral inhibition, behavioral inhibition and distal defense strategies, and COVID-19 threat levels and distal defense strategies when controlling for its indirect association with behavioral inhibition. Given these effect sizes, a sample size of *N* = 453 was required to detect the indirect association of perceived threat through COVID-19 and the distal defense strategies over behavioral inhibition with a likelihood of 80%. We decided to recruit 500 participants^4^ to account for possible exclusions and to compensate for dropouts.

##### Exclusions

Nine participants that did not complete the survey and/or showed a suspicious response pattern in the questionnaires by consistently ticking the same answer or Likert scale point were excluded. Furthermore, 147 participants were excluded because they failed an attention check (“please tick ‘not at all’ here”), which was implemented in the outgroup derogation assessment. Unexpectedly, a substantial percentage of the remaining sample (27.74%, 118 people) did not state American as their nationality. Since the group-related defense strategies used in this study were tailored toward United States-based party affiliation, we decided to exclude these participants as well (this exclusion criterion was not preregistered). One participant gave incoherent answers to the political goals questionnaire and was excluded from analyses as well. The final sample size for this study therefore was *N*_*final*_ = 358. Hence, our study was slightly underpowered based on the power analysis described before. Of our final sample, 197 participants identified themselves as male, whereas 159 participants identified themselves as female. Two participants did not identify themselves with any of the above. Mean age was *M* = 40.61, *SD* = 12.80.

#### Procedure

Participants were first asked to indicate their level of perceived threat due to the pandemic and then filled in a behavioral inhibition scale. Afterward, they completed several scales that assessed their use of the following distal defense strategies: passive party support, control restoration motivation, ingroup bias, ingroup entitativity, and outgroup derogation. Finally, participants’ demographic information was assessed.

#### Measures

##### COVID-19 threat scale

We assessed perceived COVID-19 threat with the newly developed COVID-19 Threat Scale (sample item: “Because of the Coronavirus, what happens in my life is currently beyond my control”) that incorporates the four main threats (uncertainty, violation of expectancies, lack of autonomy, and lack of agency) of the virus (see Appendix A for a full list of the scale’s items, Reiss et al., under review). Participants responded using a six-point Likert scale ranging from “strongly disagree” to “strongly agree.” Factor analysis during scale development has suggested that the items that register violation of expectancies (i.e., epistemic discrepancies) represent an own factor next to the one formed by the rest of the scale (Reiss et al., under review). In the present sample, excluding the three epistemic discrepancy items (e.g., “The Corona pandemic surprised me”) increased Cronbach’s alpha from α = 0.81 to α = 0.84. Hence, analyses were conducted with a revised COVID-19 Threat Scale not including the three epistemic discrepancy items. By doing so, we deviated from our preregistered analysis plan according to which the full COVID-19 Threat Scale would have been used. The subsequent results differ only marginally for the full versus the revised COVID-19 Threat Scale. The results for Study 1 when using the full COVID-19 Threat Scale can be found in Appendix B.

##### Behavioral inhibition

The level of activation of the BIS was assessed with eight items asking participants how afraid, scared, frightened, nervous, jittery, shaky, inhibited, and worried they felt; Cronbach’s alpha was excellent, α = 0.95. Answers were given on a five-point Likert scale ranging from “disagree” to “agree.” Adjectives correspond to the subscales “fear” plus the items “inhibited” and “worried” (Agroskin et al., 2016) of the “Positive and Negative Affect Schedule – X” (PANAS-X; Watson and Clark, 1999).

##### Party affiliation/passive party support/control restoration motivation

These dependent measures were adapted from an earlier study by Fritsche et al. (2008): Participants first indicated their party affiliation (i.e., Republican or Democrat). This self-reported ingroup was then used to infer group-related defense strategies in the form of passive party support, control restoration motivation, and ingroup bias.

Participants’ passive party support was assessed *via* three questions adapted to their previously reported party affiliation: “How important would it be for you to listen to or watch an appearance of the presidential candidate of the Democratic/Republican Party on television or social media?”; “How much would you like being addressed by a representative of the Democratic/Republican Party in front of an election booth on the street?”; and “How much would you like to use a pencil with the Democratic/Republican Party’s logo at your workplace/at the university?.” The scale was a 10-point Likert scale ranging from “not at all” to “very much.” Cronbach’s alpha was good, α = 0.84.

Control restoration motivation was assessed with the single question “If you were to support the Democratic/Republican Party, would you have a feeling of ‘together we are strong’?” using the same 10-point Likert scale as for the passive party support items.

##### Ingroup bias

Ingroup bias was assessed by measuring the warmth and competence level the participants perceive for their affiliated party as well as for the opposing party. Warmth was assessed *via* the extent to which participants assign two characteristics (“warm,” “good-natured”) to the two parties. Competence was assessed *via* the extent to which participants assign two characteristics (“competent,” “intelligent”) to the parties. The mean score of the warmth (*r*s ≥ 0.86, *p*s ≤ 0.01) and competence items (*r*s ≥ 0.76, *p*s ≤ 0.01) for the non-affiliated party was then subtracted from the mean score of the warmth (*r*s ≥ 0.71, *p*s ≤ 0.01) and competence (*r*s ≥ 0.75, *p*s ≤ 0.01) items for the affiliated party to create the ingroup bias score (i.e., higher values indicate greater pro-ingroup bias). This measure was adapted from the original version by Fritsche et al. (2008).

##### Ingroup entitativity

Ingroup entitativity was assessed *via* the extent to which participants agree with two statements about their affiliated party: “Democrats/Republicans share a common nature” and “Democrats/Republicans share common goals and a common fate” (*r*s ≥ 0.75, *p*s ≤ 0.01). Answers were given on a 10-point Likert scale ranging from “not at all” to “very much.”

##### Outgroup derogation

Outgroup derogation was assessed by asking participants to which extent they agree to the following statements about the opposing party: “I would accept a Democrat/Republican working with me”; “I would have nothing against a Democrat/Republican moving into the neighboring apartment/house”; “I would not mind a Democrat/Republican marrying a member of my family”; “I have positive feelings toward Democrats/Republicans”; and “I fully trust Democrats/Republicans.” Answers were given on a 10-point Likert scale ranging from “not at all” to “very much.” Cronbach’s alpha was good, α = 0.81. This measure was adapted from the original version by Fritsche et al. (2008).

#### Results

For an overview of correlation coefficients between all measures, see Table 1. As expected, COVID-19 threat levels were associated with greater activation of the BIS, *r*(356) = 0.62, *p* < 0.001, 95% CIs [0.54, 0.70]. COVID-19 threat levels were also positively associated with ingroup entitativity, *r*(356) = 0.12, *p* = 0.021, 95% CIs [0.02, 0.23]; control restoration motivation, *r*(356) = 0.12, *p* = 0.025, 95% CIs [0.02, 0.22]; and passive party support, *r*(356) = 0.12, *p* = 0.028, 95% CIs [0.01, 0.22]. No significant correlation emerged between COVID-19 threat levels and ingroup bias, *r*(356) = 0.08, *p* = 0.123, 95% CIs [−0.02, 0.19], as well as outgroup derogation *r*(356) = −0.03, *p* = 0.625, 95% CIs [−0.13, 0.08].

**TABLE 1:** Correlation matrix (*n* = 358).

Variable	1	2	3	4	5	6	7
1. COVID-19 threat level	–	0.62**	0.12*	0.12*	0.08	0.12*	−0.03
2. BIS		–	0.18**	0.20**	0.04	0.24**	0.03
3. Ingroup entitativity			–	0.69**	0.34**	0.56**	0.02
4. Control restoration motivation				–	0.38**	0.73**	−0.04
5. Ingroup bias					–	0.25**	−0.48**
6. Passive party support						–	−0.04
7. Outgroup derogation							–
*M*	3.73	2.89	7.34	7.06	1.91	6.26	6.28
*SD*	0.83	1.2	1.88	2.51	1.91	2.44	2.11
Range possible	1–6	1–5	1–10	1–10	−6 to 6	1–10	1–10
Range actual	1–5.83	1–5	1–10	1–10	−2.75 to 6	1–10	1–10

*p < 0.05 (two-tailed). **p < 0.01 (two-tailed).

To test the hypothesized indirect associations between COVID-19 threat levels and the defense strategies over behavioral inhibition, we ran simple mediation analyses *via* Hayes’ SPSS PROCESS macro (version 3.4) separately for each of the defense variables that were found to correlate positively with the activation level of the BIS. This was the case for ingroup entitativity, *r*(356) = 0.18, *p* = 0.001, 95% CIs [0.08, 0.28]; control restoration motivation, *r*(356) = 0.20, *p* < 0.001, 95% CIs [0.09, 0.30]; and passive party support, *r*(356) = 0.24, *p* < 0.001, 95% CIs [0.14, 0.34]. Behavioral inhibition did not correlate significantly with ingroup bias, *r*(356) = 0.04, *p* = 0.411, 95% CIs [−0.06, 0.15], nor with outgroup derogation, *r*(356) = 0.03, *p* = 0.534, 95% CIs [−0.07, 0.14].

For each mediation analysis, 5,000 bootstrap samples were created to establish a 95% bias-corrected confidence interval for the expected indirect associations^5^.

##### Main analyses

**FIGURE 1:**
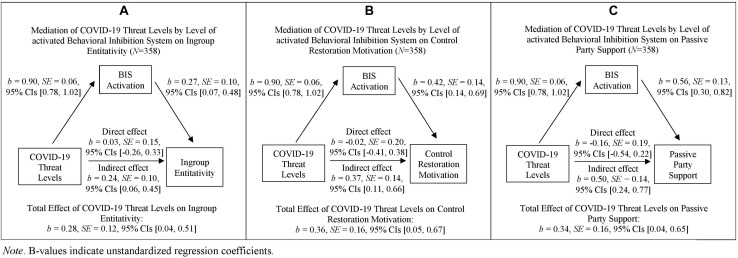
Main analyses: Simple mediation analyses for the distal defenses of Ingroup Entitativity **(A)**, Control Restoration Motivation **(B)**, and Passive Party Support **(C)**. *B*-values indicate unstandardized regression coefficients.

Mediation analyses showed that the expected indirect effects of COVID-19 threat on distal defense strategies through BIS were significant for ingroup entitativity, control restoration motivation, and passive party support. Indirect effects were not significant for ingroup bias, *b* = −0.02, *SE* = 0.10, 95% CIs [−0.20, 0.17], and outgroup derogation, *b* = 0.13, *SE* = 0.12, 95% CIs [−0.11, 0.35]. Detailed statistical values for the significant indirect effects are presented for each dependent variable (DV) separately in the following (see also Figure 1).

###### Ingroup entitativity

The regression coefficient between COVID-19 threat levels and ingroup entitativity was significant (total effect: *b* = 0.28, *SE* = 0.12, *t*(356) = 2.31, *p* = 0.021, 95% CIs [0.04, 0.51]). Importantly and as predicted, the indirect effect of COVID-19 threat levels *via* behavioral inhibition levels on ingroup entitativity was significant, *b* = 0.24, *SE* = 0.10, 95% CIs [0.06, 0.45]. Furthermore, the regression coefficient between behavioral inhibition levels and ingroup entitativity when controlling for the variance shared by COVID-19 threat levels and ingroup entitativity was significant, *b* = 0.27, *SE* = 0.10, *t*(356) = 2.59, *p* = 0.010, 95% CIs [0.07, 0.48]. The direct effect of COVID-19 threat levels on ingroup entitativity when controlling for the variance shared by behavioral inhibition level and ingroup entitativity was non-significant, *b* = 0.03, *SE* = 0.15, *t*(356) = 0.22, *p* = 0.829, 95% CIs [−0.26, 0.33], see also Figure 1A.

###### Control restoration motivation

The total effect of COVID-19 threat levels on control restoration motivation was significant, *b* = 0.36, *SE* = 0.16, *t*(356) = 2.25, *p* = 0.025, 95% CIs [0.05, 0.67]. Importantly and as predicted, the indirect effect of COVID-19 threat levels *via* behavioral inhibition levels on control restoration motivation was significant, *b* = 0.37, *SE* = 0.14, 95% CIs [0.11, 0.66]. Furthermore, the regression coefficient between behavioral inhibition levels and control restoration motivation when controlling for the variance shared by COVID-19 threat levels and control restoration motivation was significant, *b* = 0.42, *SE* = 0.14, *t*(356) = 3.00, *p* = 0.003, 95% CIs [0.14, 0.69]. The direct effect of COVID-19 threat levels on control restoration motivation was non-significant, *b* = −0.02, *SE* = 0.20, *t*(356) = 0.08, *p* = 0.938, 95% CIs [−0.41, 0.38], see also Figure 1B.

###### Passive party support

The regression coefficient between COVID-19 threat levels and passive party support (i.e., total effect) was significant, *b* = 0.34, *SE* = 0.16, *t*(356) = 2.21, *p* = 0.028, 95% CIs [0.04, 0.65]. Importantly and as predicted, the indirect effect of COVID-19 threat levels *via* behavioral inhibition levels on passive party support was significant, *b* = 0.50, *SE* = 0.14, 95% CIs [0.24, 0.77]. The regression coefficient between behavioral inhibition levels and passive party support when controlling for the variance shared by COVID-19 threat levels and passive party support was significant, *b* = 0.56, *SE* = 0.13, *t*(356) = 4.20, *p* < 0.001, 95% CIs [0.30, 0.82]. The direct effect of COVID-19 threat levels on passive party support was non-significant, *b* = −0.16, *SE* = 0.19, *t*(356) = 0.84, *p* = 0.402, 95% CIs [−0.54, 0.22], see also Figure 1C.

##### Exploratory analyses

**FIGURE 2:**
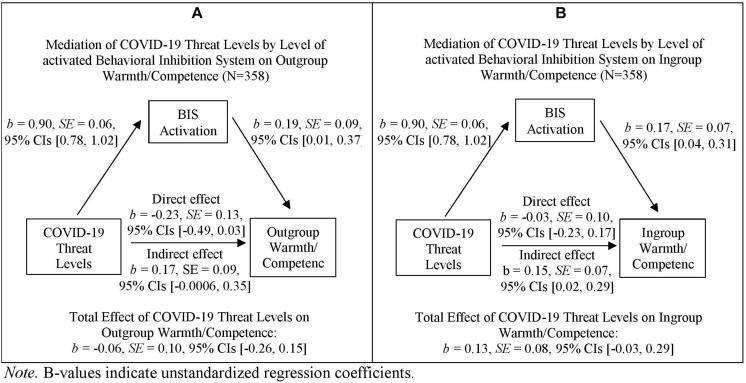
Simple mediation analyses for Outgroup Warmth/Competence **(A)**, and Ingroup Warmth/Competence **(B)**. *B*-values indicate unstandardized regression coefficients.

In order to better understand the non-significant correlation between activation levels of the BIS and ingroup bias, we ran exploratory mediation analyses for the two components of ingroup bias, namely outgroup warmth/competence rating and ingroup warmth/competence rating (see also Figure 2).

###### Outgroup warmth/competence

The regression coefficient between COVID-19 threat levels and outgroup warmth/competence rating (i.e., total effect) was non-significant, *b* = −0.06, *SE* = 0.10, *t*(356) = 0.57, *p* = 0.572, 95% CIs [−0.26, 0.15]. The indirect effect of COVID-19 threat levels *via* behavioral inhibition levels on outgroup warmth/competence rating was marginally significant, *b* = 0.17, *SE* = 0.09, 95% CIs [−0.0006, 0.35].

Furthermore, the regression coefficient between behavioral inhibition levels and outgroup warmth/competence rating when controlling for the variance shared by COVID-19 threat levels and outgroup warmth/competence was significant, *b* = 0.19, *SE* = 0.09, *t*(356) = 2.12, *p* = 0.034, 95% CIs [0.01, 0.37]. The direct effect, namely the regression coefficient between COVID-19 threat levels and outgroup warmth/competence rating, was marginally significant, *b* = −0.23, *SE* = 0.13, *t*(356) = −1.76, *p* = 0.079, 95% CIs [−0.49, 0.03], see also Figure 2A.

###### Ingroup warmth/competence

The regression coefficient between COVID-19 threat levels and ingroup warmth/competence rating (i.e., total effect) was non-significant, *b* = 0.13, *SE* = 0.08, *t*(356) = 1.63, *p* = 0.105, 95% CIs [−0.03, 0.29]. The indirect effect of COVID-19 threat levels *via* behavioral inhibition levels on ingroup warmth/competence rating was significant, *b* = 0.15, *SE* = 0.07, 95% CIs [0.02, 0.29]. Furthermore, the regression coefficient between behavioral inhibition levels and ingroup warmth/competence rating when controlling for the variance shared by COVID-19 threat levels and ingroup bias was significant, *b* = 0.17, *SE* = 0.07, *t*(356) = 2.51, *p* = 0.013, 95% CIs [0.04, 0.31]. The direct effect, namely the regression coefficient between COVID-19 threat levels and ingroup warmth/competence rating, was non-significant, *b* = −0.03, *SE* = 0.10, *t*(356) = 0.27, *p* = 0.783, 95% CIs [−0.23, 0.17], see also Figure 2B.

#### Discussion

Confirming H1, H2, and H4, we found significant positive correlations between COVID-19 threat levels and ingroup entitativity, control restoration motivation, and passive party support. Contrary to our predictions (i.e., H3 and H5), no positive correlation between COVID-19 threat levels and ingroup bias as well as outgroup derogation emerged. A possible explanation for these null findings is given below in the discussion of the expected mediated effects between COVID-19 threat levels and distal defenses *via* heightened behavioral inhibition. Hence, three of the five preregistered main effects of Study 1 could be confirmed.

Confirming H6, H7, and H9, we found indirect effects indicating that people who perceived greater COVID-19 threat showed greater ingroup entitativity, control restoration motivation, and passive party support as a result of a heightened BIS activation. These results support our argument that people’s responses to COVID-19 can, at least in part, be explained by the proposed motivational process of the General Process Model of Threat and Defense.

The expected indirect associations for outgroup derogation and ingroup bias were non-significant, and thus, H8 and H10 were not supported. A possible reason for these null results might be the strong national identity of the United States population used in this study: When feeling threatened by COVID-19, participants might not only turn to their political but also to their national ingroup. Hence, they may have shown increased ingroup favoritism regarding their own political ingroup but no outgroup derogation regarding their political outgroup since the members of this outgroup are still part of the national ingroup. This would be in line with research showing that indicators of political outgroup derogation are mitigated when United States participants are reminded of their American identity (Levendusky, 2018). Support for this explanation also derives from the fact that we found neither a positive correlation between COVID-19 threat levels and outgroup derogation nor a negative correlation between COVID-19 threat levels and the combined warmth/competence rating of the political outgroup. Instead, we actually found a positive indirect association of COVID-19 threat levels and ingroup as well as outgroup warmth/competence rating *via* BIS with the latter indirect association being only marginally significant. This suggests that under heightened perceived threat through COVID-19, United States citizens see their own as well as the opposing political party more positively. To sum up, three of the five preregistered mediated effects of Study 1 could be confirmed.

### Study 2

Since Study 1 was of correlational nature, it did not allow to test for the hypothesized causal effects of COVID-19 threat levels over heightened activation of the BIS on distal defense strategies. Hence, in Study 2, a COVID-19 threat manipulation was introduced which aimed to increase versus decrease the salience of COVID-19 threat. As in Study 1, Study 2 was a Qualtrics online study and links were sent to a United States-based MTurk workers sample (*N*_*total*_ = 648) *via* Amazon’s Mechanical Turk.

#### Participants and Exclusions

##### Sample size

The sample size of Study 2 was determined beforehand *via* a power analysis designed to find the hypothesized indirect effect with a likelihood of 80%, setting alpha error probability to α = 0.05. The power analysis was conducted with Kenny (2017) MedPower application. We assumed an effect size of *r* = 0.15 for the effect of the COVID-19 threat manipulation on behavioral inhibition, the effect of behavioral inhibition on the DVs, and the effect of the COVID-19 threat manipulation on the DVs when controlling for the effect of behavioral inhibition on the DVs. Given these effect sizes, a sample size of *N* = 453 was required to detect the indirect effect of the COVID-19 threat manipulation on the DVs over behavioral inhibition with a likelihood of 80%. We decided to recruit 500 participants^6^ to account for possible exclusions and to compensate for dropouts.

##### Exclusions

One hundred and twenty participants who did not complete the survey and/or showed a suspicious response pattern in the questionnaires by consistently ticking the same answer were excluded. Additionally, 39 participants failing the attention check (“Please ignore the question and only write down the word football into the box as the answer to the question”) were excluded. As in Study 1, a substantial percentage of the remaining sample (27.8%, *N* = 134) did not state American as their nationality. Since the group-related defense strategies used in this study were tailored toward United States citizens, we again decided to exclude these participants (this exclusion criteria was not preregistered), as well as seven participants that additionally did not give coherent answers for the COVID-19 manipulation. The final sample size for this study therefore was *N*_*final*_ = 348. Hence, our study was slightly underpowered based on the power analysis described before. Of our final sample, 204 participants identified themselves as male, whereas 142 participants themselves identified as female. Two participants did not identify themselves with any of the above. Mean age was *M* = 37.73, *SD* = 10.81.

#### Procedure

After manipulating COVID-19 threat salience, we measured participants’ perceived COVID-19 threat salience as a manipulation check by asking them to what extent they agree with the following two statements: “I think the facts displayed were potentially threatening” and “After reading these facts I feel relaxed” (reverse coded). Answers were given on a 10-point Likert scale ranging from “Not at all” to “A lot.” We then assessed participants’ activation of the BIS and the BAS. Finally, we assessed their use of defense strategies as indexed by ingroup bias, the perceived entitativity of citizens of the United States, system justification tendencies, and their belief in two corona-related conspiracies.

**FIGURE 3:**
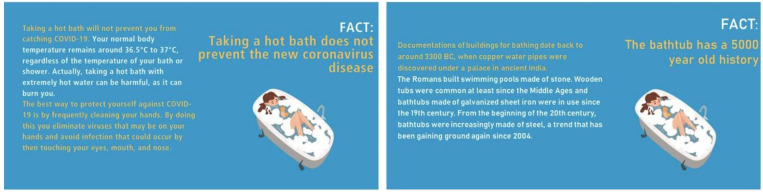
Mythbusters for the threat **(left)** and the control condition **(right)**.

To manipulate perceived COVID-19 threat salience, participants had to answer questions regarding several mythbusters (World Health Organisation [WHO], 2020) that either concluded that there is no cure available to COVID-19 (i.e., threat condition) or contained information completely unrelated to COVID-19 (i.e., control condition; see Figure 3). Answering these questions was meant to either remind participants of the threats caused by COVID-19 or to direct their attention away from them.

#### Measures

##### Behavioral inhibition/behavioral approach

Participants then completed the same BIS assessment used in Study 1; Cronbach’s alpha was excellent, α = 0.94. For exploratory reasons, we also included an assessment of participants’ activation level of the BAS; Cronbach’s alpha was good, α = 0.87. It was assessed by asking participants to what extent 10 adjectives (i.e., active, alert, attentive, determined, enthusiastic, excited, inspired, proud, strong, interested) represent what they currently feel (taken from the subscale “Positive Affect,” PANAS-X; Watson and Clark, 1999; Stollberg et al., under review).

##### Ingroup bias

The assessment of participants’ level of ingroup bias and ingroup entitativity was identical to that of Study 1, except for the difference that the relevant ingroup was not the participants’ political affiliation but their nationality (i.e., United States-American). The mean score of the warmth (*r*(346) = 0.77, *p* ≤ 0.001, 95% CIs [0.70, 0.84]) and competence items (*r*(346) = 0.78, *p* ≤ 0.001, 95% CIs [0.71, 0.84]) for the outgroup was once again subtracted from the mean score of the warmth (*r*(346) = 0.73, *p* ≤ 0.001, 95% CIs [0.66, 0.80]) and competence (*r*(346) = 0.83, *p* ≤ 0.001, 95% CIs [0.77, 0.89]) items for the ingroup to create the ingroup bias score (i.e., higher values indicate greater pro-ingroup bias). The relevant outgroup the participants had to judge regarding warmth and competence was Chinese citizens. We chose this outgroup since conspiratorial beliefs were being shared that claimed the virus was intentionally designed by China (Gertz, 2020). Furthermore, an increase of critique and negative perception of China could be observed in the United States since the beginning of the pandemic (Silver, 2020).

##### System justification

Afterward, we assessed participants’ tendency to justify the systems they live in as well as the degree they perceived the measures taken against COVID-19 as justified. The System Justification Scale by Kay and Jost (2003) was adopted to the pandemic for this purpose. Answers to eight statements (sample item: “I find our society fair when combating the coronavirus”; Cronbach’s alpha was good, α = 0.83) were given on a seven-point Likert scale ranging from “I don’t agree at all” to “I very much agree.”

##### Belief in conspiratorial ideas

To infer the level of belief in conspiracies, participants were confronted with conspiratorial claims about the virus in two scenarios such as “COVID-19 was developed as a biological weapon by the Chinese government. Due to a laboratory accident, it was spread among China’s own population” and had to rate to what extent they believed the claims were true on a five-point Likert scale ranging from “definitely not true” to “definitely true,” *r*(246) = 0.25, *p* ≤ 0.001, 95% CIs [0.14, 0.35].

#### Results

##### Manipulation check

**FIGURE 4:**
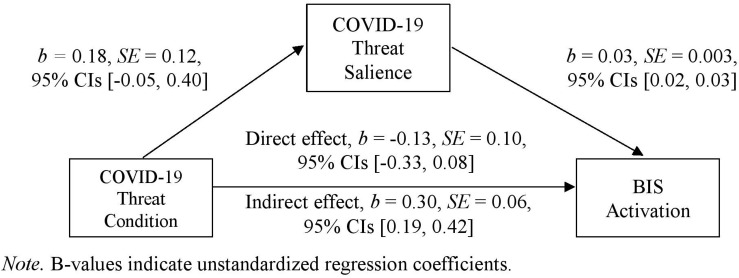
Mediation of COVID-19 threat condition by COVID-19 threat salience on behavioral inhibition (*N* = 348). *B*-values indicate unstandardized regression coefficients.

A simple mediation analysis *via* Hayes’ SPSS PROCESS macro (version 3.4) showed a significant indirect effect of the COVID-19 threat manipulation on the activation of the BIS as a result of increased perceived COVID-19 threat salience, *b* = 0.30, *SE* = 0.06, 95% CIs [0.19, 0.42], see Figure 4^7^. The COVID-19 threat manipulation did not directly affect participants’ activation of the BIS in the threat condition (*M* = 2.31, *SD* = 1.07) compared with the control condition (*M* = 2.13, *SD* = 1.08, *t*(346) = 1.53, *p* = 0.127, *d* = 0.17, 95% CIs [−0.40, 0.05]) even though the effect went into the right direction. The COVID-19 threat manipulation also did not directly affect the distal defenses. The significant indirect effect shows that even if the COVID-19 threat manipulation did not directly affect BIS, the COVID-19 threat manipulation worked in so far as it increased activation levels of the BIS over increased perceived COVID-19 threat salience (for an overview of correlation coefficients between all measures, see Table 2).

**TABLE 2:** Correlation matrix (*n* = 348).

Variable	1	2	3	4	5	6	7	8
1. COVID-19 Threat Condition	–	0.28**	0.08	−0.14*	−0.05	−0.04	−0.07	−0.01
2. Perceived COVID-19 Threat Salience		–	0.50**	−0.23**	−0.07	−0.09	0.16**	−0.02
3. BIS			–	0.07	−0.03	0.08	0.30**	0.14*
4. BAS				–	0.16**	0.45**	0.22**	0.39**
5. Ingroup bias					–	0.32**	0.27**	0.36**
6. Entitativity						–	0.23**	0.55**
7. Conspiracy Beliefs							–	0.37**
8. System Justification								
*M*	N/A	−15.95	2.22	3.4	0.29	7.02	3.21	4.02
*SD*	N/A	20.78	1.08	0.83	1.28	2.13	0.96	1.21
Range possible	N/A	−50 to 50	1–5	1–5	−6 to 6	1–10	1–5	1–7
Range actual	N/A	−50 to 50	1–4.63	1–5	−2.5 to 6	1–10	1–5	1–7

*p < 0.05 (two-tailed). **p < 0.01 (two-tailed).

##### Serial mediation analyses

**FIGURE 5:**
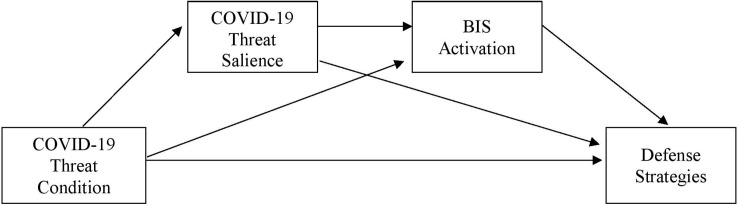
Serial mediation of COVID-19 threat condition by COVID-19 threat salience and BIS activation on defense strategies.

To test for the hypothesized causal effects of the COVID-19 threat manipulation over heightened activation of the BIS and increased perceived COVID-19 threat salience on distal defense strategies, serial mediations with threat condition as the predictor variable and perceived COVID-19 threat salience and activation of the BIS as the mediators were run for each of the dependent variables (see Figure 5).

For ingroup entitativity, system justification, and conspiracy beliefs, a significant positive indirect effect of threat condition over the two mediators was found: *b* = 0.10, *SE* = 0.05, 95% CIs [0.02, 0.19]; *b* = 0.07, *SE* = 0.03, 95% CIs [0.02, 0.12]; and *b* = 0.08, *SE* = 0.02, 95% CIs [0.04, 0.13], respectively. The indirect effect remained non-significant for ingroup bias, *b* = −0.003, *SE* = 0.02, 95% CIs [−0.04, 0.05].

##### Simple mediation analyses

In the preregistration of Study 2, we stated that independent sample *t*-tests would be run to investigate the association between the COVID-19 threat manipulation and the dependent variables. Furthermore, we stated that the expected indirect positive effect of the COVID-19 threat manipulation *via* behavioral inhibition on the outlined dependent variables would be tested. Since the manipulation check revealed a non-significant correlation between the COVID-19 threat manipulation and the activation level of the BIS as well as the dependent variables, we neither ran the preregistered *t*-test analyses nor the simple mediation analyses to test for the effect of the COVID-19 threat manipulation over activation of the BIS on the dependent variables. Instead, we ran simple mediation analyses to test for the indirect effect of perceived COVID-19 threat salience over activation of the BIS on the dependent variables.

###### Ingroup entitativity

**FIGURE 6:**
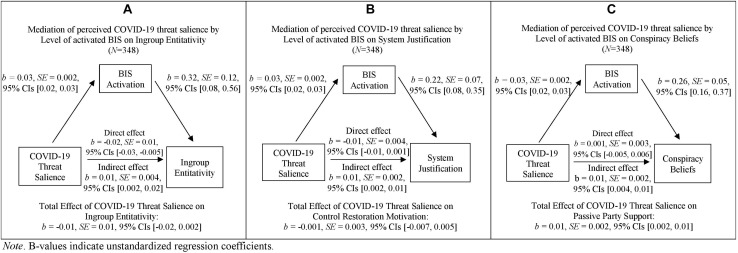
Simple mediation analyses for the indirect effect of COVID-19 threat salience via BIS activation on distal defenses of Ingroup Entitativity **(A)**, System Justification **(B)**, and Conspiracy Beliefs **(C)**. *B*-values indicate unstandardized regression coefficients.

The regression coefficient between perceived COVID-19 threat salience and ingroup entitativity was non-significant, *b* = −0.01, *SE* = 0.01, *t*(346) = 1.61, *p* = 0.108, 95% CIs [−0.02, 0.002], i.e., total effect. The indirect effect of perceived COVID-19 threat salience *via* behavioral inhibition levels on ingroup entitativity was significant, *b* = 0.01, *SE* = 0.004, 95% CIs [0.002, 0.02]. Furthermore, the regression coefficient between behavioral inhibition levels and ingroup entitativity when controlling for the variance shared by perceived COVID-19 threat salience and ingroup entitativity was significant, *b* = 0.32, *SE* = 0.12, *t*(346) = 2.65, *p* = 0.008, 95% CIs [0.08, 0.56]. The direct effect, namely the regression coefficient between perceived COVID-19 threat salience and ingroup entitativity, was significant, *b* = −0.02, *SE* = 0.01, *t*(346) = 2.72, *p* = 0.007, 95% CIs [−0.03, −0.005]. Unexpectedly, the regression coefficient was negative, indicating that higher threat salience led to lower ingroup entitativity when controlling for the effect of participants’ activation level of the BIS (see Figure 6A).

###### System justification

The regression coefficient between perceived COVID-19 threat salience and system justification was non-significant, *b* = −0.001, *SE* = 0.003, *t*(346) = 0.40, *p* = 0.690, 95% CIs [−0.007, 0.005], i.e., total effect. The indirect effect of perceived COVID-19 threat salience *via* behavioral inhibition levels on system justification was significant, *b* = 0.01, *SE* = 0.002, 95% CIs [0.002, 0.01]. Furthermore, the regression coefficient between behavioral inhibition levels and system justification when controlling for the variance shared by perceived COVID-19 threat salience and system justification was significant, *b* = 0.22, *SE* = 0.07, *t*(346) = 3.20, *p* = 0.002, 95% CIs [0.08, 0.35]. The direct effect, namely the regression coefficient between perceived COVID-19 threat salience and system justification, was marginally significant, *b* = −0.01, *SE* = 0.004, *t*(346) = 1.93, *p* = 0.054, 95% CIs [−0.01, 0.0001]. Unexpectedly, the regression coefficient was negative, indicating that higher COVID-19 threat salience led to lower system justification when controlling for the effect of participants’ activation level of the BIS (see Figure 6B).

###### Conspiracy beliefs

The regression coefficient between perceived COVID-19 threat salience and conspiracy beliefs (i.e., total effect) was significant, *b* = 0.01, SE = 0.002, *t*(346) = 2.95, *p* = 0.003, 95% CIs [0.002, 0.01]. The indirect effect of perceived COVID-19 threat salience *via* behavioral inhibition levels on conspiracy beliefs was significant, *b* = 0.01, *SE* = 0.002, 95% CIs [0.004, 0.01]. Furthermore, the regression coefficient between behavioral inhibition levels and conspiracy beliefs when controlling for the variance shared by perceived COVID-19 threat salience and conspiracy beliefs was significant, *b* = 0.26, *SE* = 0.05, *t*(346) = 5.02, *p* < 0.001, 95% CIs [0.16, 0.37]. The direct effect, namely the regression coefficient between perceived COVID-19 threat salience and conspiracy beliefs, was non-significant, *b* = 0.001, *SE* = 0.003, *t*(346) = 0.17, *p* = 0.865, 95% CIs [−0.005, 0.006] (see Figure 6C).

###### Ingroup bias

The regression coefficient between perceived COVID-19 threat salience and ingroup bias (i.e., total effect) was non-significant, *b* = −0.004, *SE* = 0.003, *t*(346) = 1.25, *p* = 0.213, 95% CIs [−0.01, 0.002]. The indirect effect of perceived COVID-19 threat salience *via* behavioral inhibition levels on ingroup bias was also non-significant, *b* < 0.001, *SE* = 0.002, 95% CIs [−0.003, 0.004]. Furthermore, the regression coefficient between behavioral inhibition levels and ingroup bias when controlling for the variance shared by perceived COVID-19 threat salience and ingroup bias was non-significant, *b* = 0.002, *SE* = 0.07, *t*(346) = 0.03, *p* = 0.980, 95% CIs [−0.14, 0.15]. The direct effect, namely the regression coefficient between perceived COVID-19 threat salience and ingroup bias, was non-significant, *b* = −0.004, *SE* = 0.004, *t*(346) = 1.09, *p* = 0.275, 95% CIs [−0.01, 0.003].

As in Study 1 and in order to better understand the non-significant effect of perceived COVID-19 threat salience on ingroup bias, we ran exploratory mediation analyses for the two components of ingroup bias, namely outgroup warmth/competence rating and ingroup warmth/competence rating.

The regression coefficient between perceived COVID-19 threat salience and outgroup warmth/competence rating (i.e., total effect) was non-significant, *b* = −0.004, *SE* = 0.003, *t*(346) = 1.11, *p* = 0.267, 95% CIs [−0.011, 0.003]. The indirect effect of perceived COVID-19 threat salience *via* behavioral inhibition levels on outgroup warmth/competence rating was non-significant, *b* = 0.003, *SE* = 0.002, 95% CIs [−0.001, 0.007]. Furthermore, the regression coefficient between behavioral inhibition levels and outgroup warmth/competence rating when controlling for the variance shared by perceived COVID-19 threat salience and outgroup warmth/competence was non-significant, *b* = 0.12, *SE* = 0.08, *t*(346) = 1.54, *p* = 0.126, 95% CIs [−0.03, 0.27]. The direct effect, namely the regression coefficient between perceived COVID-19 threat salience and outgroup warmth/competence rating, was marginally significant, *b* = −0.01, *SE* = 0.004, *t*(346) = 1.73, *p* = 0.085, 95% CIs [−0.015, 0.001].

The regression coefficient between perceived COVID-19 threat salience and ingroup warmth/competence rating (i.e., total effect) was significant, *b* = −0.01, *SE* = 0.003, *t*(346) = 2.45, *p* = 0.015, 95% CIs [−0.014, −0.002]. The indirect effect of perceived COVID-19 threat salience *via* behavioral inhibition levels on ingroup warmth/competence rating was non-significant, *b* = 0.003, *SE* = 0.002, 95% CIs [−0.001, 0.007]. Furthermore, the regression coefficient between behavioral inhibition levels and ingroup warmth/competence rating when controlling for the variance shared by perceived COVID-19 threat salience and ingroup bias was non-significant, *b* = 0.12, *SE* = 0.07, *t*(346) = 1.65, *p* = 0.100, 95% CIs [−0.02, 0.26]. The direct effect, namely the regression coefficient between perceived COVID-19 threat salience and ingroup warmth/competence rating, was significant, *b* = −0.011, *SE* = 0.004, *t*(346) = 2.95, *p* = 0.003, 95% CIs [−0.018, −0.004].

#### Discussion

Study 2 allowed for testing a causal relationship between participants’ perceived COVID-19 threat salience and increased use of distal defenses *via* higher activation levels of the BIS. Results showed that the COVID-19 threat manipulation did not directly affect the activation of the BIS as well as the use of distal defense strategies. Thus, the preregistered hypotheses H1 to H4 suggesting the main effects of the COVID-19 threat manipulation onto distal defenses as well as hypotheses H5 to H8 suggesting the mediation effects of the COVID-19 threat manipulation onto distal defenses *via* heightened activation of the BIS could not be confirmed. However, participants in the high-threat condition indicated to feel more threatened by COVID-19 than the control group, and the experienced COVID-19 threat was associated with greater activation of the BIS. Findings further showed that greater perceived threat was associated with greater use of distal defense strategies as a result of greater activation of the BIS. Specifically, we found significant indirect effects of the COVID-19 threat manipulation on the distal defenses ingroup entitativity, system justification, and conspiracy beliefs serially mediated by participants’ perceived COVID-19 threat salience and therefore increased activation of their BIS.

These findings are in line with the hypothesis that an increase in people’s perceived COVID-19 threat level induces a proximal threat reaction in the form of an increased activation level of the BIS which in turn, is associated with a heightened engagement with distal defenses in order to lower the activation level of the BIS. Unexpectedly, perceived COVID-19 threat had a negative direct effect on ingroup entitativity and system justification. To explain these results, it is important to point out that COVID-19 threat levels and activation levels of the BIS shared a substantial amount of variance (*R*^2^ = 0.245) suggesting multicollinearity. Hence, the significant negative regression coefficients of COVID-19 threat levels might be due to the missing shared variance between activation levels of the BIS and the defense strategies ingroup entitativity and system justification (Belsley, 1991). Supporting this explanation is the fact that – without activation levels of the BIS as a covariate – the correlations between the two defense strategies and COVID-19 threat levels remained non-significant (see Table 2).

Results also yielded positive correlations between participants’ activation level of the BAS and the dependent variables (see Table 2). These results can be explained by the General Process Model of Threat and Defense as well: The model suggests that increased approach motivation as part of the activation of the BAS enhances the use of defensive strategies. In concrete, high behavioral inhibition – which requires the use of defense strategies – and behavioral approach – which motivates to approach the use of defenses – might interact insofar, as high inhibition coupled with high behavioral activation leads to increased defensive reactions (Klackl et al., under review). This means that participants who had a higher activation level of behavioral approach when being confronted with the COVID-19 threat manipulation might have shown increased use of defense strategies because they were to a greater extent able to tackle the state of behavioral inhibition by approaching and using the available defenses.

## General Discussion

Van Bavel et al. (2020) recently pointed out that behavioral and social sciences are able to inform policy makers as well as the broader public to foster the positive consequences while diminishing the negative consequences of COVID-19-related reactions. In order to do so, it is essential to investigate and understand what exactly causes these reactions. The present research aimed at answering this question by proposing and testing a possible mechanism through which the manifold human reactions to COVID-19 may occur. In line with the reasoning of the General Process Model of Threat and Defense, we hypothesized COVID-19 reactions to represent distal defense strategies whose purpose it is to lower the activation level of the BIS and the associated increased levels of anxiety and vigilance. The results of two preregistered studies supported this hypothesis. The two studies showed that people who experienced greater COVID-19 threat also showed a greater activation of the BIS. This, in turn, was related to responses representing distal defense strategies.

In Study 1, increased perceived threat through COVID-19 was indirectly associated with greater ingroup entitativity, control restoration motivation, and passive party support *via* increased activation levels of the BIS. This association was not given for outgroup derogation and ingroup bias. In Study 2, experimentally increased COVID-19 threat salience was indirectly (as a result of heightened perceived threat) associated with greater activation of the BIS. This increase in turn was associated with greater ingroup entitativity, system justification, and belief in conspiracy theories.

It is notable that in both studies the proposed mediator of BIS activation was measured and not itself manipulated. This omission requires us to be careful when interpreting the mediated effects found (Green et al., 2010). It could be, for instance, that not BIS activation but a related construct such as mortality salience is the actual mediator carrying the effect of COVID-19 threat on distal defense strategies (Menzies and Menzies, 2020). In this case, activation of the BIS might just covary with the true mediator. Even though the studies of the present research cannot rule out this possibility, results are consistent with past studies showing that various threats increase the use of different distal defense strategies *via* BIS-related emotions such as anxiety. One experimental setup, for instance, found that the effect of different types of mortality salience on worldview defense was mediated by negative affect (Echebarria Echabe and Perez, 2016). Additionally, Webber et al. (2015) demonstrated the pivotal role of emotions in worldview defense in their placebo study: When participants had the chance to attribute their anxiety to something other than threat, they showed less worldview defense afterward – indicating the importance of BIS-related variables in threat processing.

### Impeding Negative Consequences

As already mentioned, many if not most of the human reactions to COVID-19 have the potential to lead to both positive and negative consequences with the negative consequences being able to have a destructive impact on individuals and society. For instance, system justification tendencies can be misused by non-democratic forms of government to extend their scope of power (Walker, 2020). Furthermore, the belief in conspiracies can entail stark detrimental consequences as well since this type of worldview defense seems to be related to diminished adherence to measures counteracting the pandemic: As Imhoff and Lamberty (2020) put it, higher belief in corona-related conspiracy theories seems to be “associated with a reduced containment-related behavior” (p. 18) regarding COVID-19 quarantine measures. Thus, taking from our results, one could argue that lowering the overall perceived COVID-19 threat levels and felt anxiety during the crisis (i.e., by media reports and political measures/messages intended to decrease anxiety) might well be an effective tool to battle the negative consequences of the reactions toward the pandemic.

While for some areas anxiety can effectively be diminished artificially (e.g., *via* media campaigns counteracting uncertainty), overall anxiety can most probably not be lowered in all domains (e.g., people will still have contact to infected individuals; hence, mortality salience will remain). This, together with the fact that not all consequences that arise as distal defenses are unambiguously negative in the first place, might offer a second strategy next to lowering anxiety overall, namely to foster the positive consequences of humans’ reactions to COVID-19.

### Fostering Positive Consequences

Positive consequences range from empowerment of democratic systems to personal prosocial actions and behavior and may hold potential for positive change. For instance, increased obedience to and acceptance of the current political system and the measures implemented by the system to tackle COVID-19 can go a long way and may well be essential to lower infection and death rates. If successful, these measures might strengthen people’s belief in democratic forms of government being able to effectively manage severe crisis – even if it means to temporarily cut people’s personal freedoms (Bol et al., 2020). Other types of threat reactions may lead to heightened support for neighbors or people of one’s own ingroup that contribute to a lasting prosocial atmosphere.

In Study 1, we found indication for increased warmth and competence ratings of both in- and outgroups under higher perceived levels of threat through COVID-19. This result might possibly be mirrored in the recent rise in support for the “Black Lives Matter” (BLM) movement by subgroups of the United States population not personally affected by systemic racism and discrimination (Arora et al., 2020). Assuming that outgroup liking (instead of outgroup derogation) can lower the threat-related consequences of COVID-19, the current research might offer an explanation for the present success of the movement. Further support for this claim provides research on the interplay of threat and prosociality: For instance, studies investigating the mortality salience threat showed that introducing participants to a prosocial norm before threat significantly increased their prosocial behavior (Jonas et al., 2008).

Prosociality and other positive consequences can not only help to cope with COVID-19 itself (as in the case of more cooperation and prosociality) but might also lead to future developments that will let us look back and see COVID-19 not only as a threat but as a chance to trigger positive change (Jutzi et al., under review).

Having provided a possible explanation of the mechanism through which COVID-19 reactions occur, future research should focus on developing measures to trigger the reactions’ positive consequences while preventing their negative ones, in order to allow us to “emerge from the crisis stronger, with better jobs and a brighter, more equal and greener future for all” (United Nations, 2020, António Guterres, UN secretary General, 19th of June, 2020).

## Data Availability Statement

The raw data supporting the conclusions of this article will be made available by the authors, without undue reservation.

## Ethics Statement

The studies involving human participants were reviewed and approved by the EK-GZ:21/2016 Ethikkommission Universität Salzburg. The patients/participants provided their written informed consent to participate in this study.

## Author Contributions

All authors listed have made a substantial, direct and intellectual contribution to the work, and approved it for publication.

## Conflict of Interest

The authors declare that the research was conducted in the absence of any commercial or financial relationships that could be construed as a potential conflict of interest.

## Footnotes

1 Links to the preregistrations: https://aspredicted.org/aa53v.pdf (Study 1) and https://aspredicted.org/y3ev9.pdf (Study 2).

2 In detail, we hypothesized a mediated association of perceived threat through COVID-19 and passive party support to participants’ preferred political party (H6), control restoration motivation (H7), ingroup bias (H8), ingroup entitativity (H9), and outgroup derogation (H10) via BIS.

3 In detail, we hypothesized a mediated effect of perceived corona threat via behavioral inhibition on ingroup bias (H5), ingroup entitativity (H6), system justification (H7), and conspiracy beliefs (H8).

4 The difference between the recruited 500 participants and the total amount of participants (*N*_*Total*_ = 633) was derived from the fact that MTurk distributed the study link to more workers than we asked for.

5 Please note that we deviated from our preregistered analysis plan, in that we did not include participants’ degree of shared goals with their affiliated party as a covariate in our analysis because this variable shared a substantial amount of variance with the DVs, hence leading to collinearity problems with our predictor variable COVID-19 threat level.

6 As in Study 1, the difference between the recruited 500 participants and the total amount of participants (*N*_*Total*_ = 648) derived from the fact that MTurk distributed the study link to more workers than we asked for.

7 For all mediation analyses in Study 25,000 bootstrap samples were created to establish a 95% bias-corrected confidence interval for the expected indirect effects.
