# Supplementary material for: Between Conspiracy Beliefs, Ingroup Bias, and System Justification: How People Use Defense Strategies to Cope With the Threat of COVID-19
Source: Front Psychol. 2020 Sep 30;11:578586. doi: 10.3389/fpsyg.2020.578586 (PMC7555435; doi:10.3389/fpsyg.2020.578586)
Supplement: Supplementary file 1 [file Data_Sheet_1.docx]

**Appendix A**

**Items of the COVID-19 Threat Scale**

Items were answered on a 6-point Likert Scale ranging from “Strongly disagree” to “Strongly agree”. Items marked with an asterisk are epistemic discrepancy items.

Despite the Coronavirus, I can do just about anything I really set my mind to. (*R*)

The Coronavirus determines most of what I can and cannot do.

Because of the Coronavirus, what happens in my life is currently beyond my control.

The Coronavirus interferes with the things I want to do.

The unpredictability of the Coronavirus outbreak does not bother me. (*R*)

During the Corona pandemic, not having all the information I need is frustrating.

I can’t stand that the Coronavirus outbreak took me by surprise.

The uncertainty surrounding the Coronavirus keeps me from living a fulfilled life.

I doubt that I can deal efficiently with unexpected consequences of the Coronavirus.

Even if I invest the necessary effort, I cannot solve the problems that arise with the Coronavirus.

I can remain calm during the Corona pandemic because I can rely on my coping abilities. (*R*)

If the Coronavirus causes problems for me, I am sure I will find a solution for them. (*R*)

The Corona pandemic surprised me.*

I expected the Corona outbreak. (*R*)*

The current Corona situation was predictable.*
