# Supplementary material for: Between Conspiracy Beliefs, Ingroup Bias, and System Justification: How People Use Defense Strategies to Cope With the Threat of COVID-19
Source: Front Psychol. 2020 Sep 30;11:578586. doi: 10.3389/fpsyg.2020.578586 (PMC7555435; doi:10.3389/fpsyg.2020.578586)
Supplement: Supplementary file 2 [file Data_Sheet_2.docx]

**Appendix B**

**Study 1 Analysis with full COVID-19 Threat Scale**

The following is the result section of Study 1 with the full COVID-19 Threat Scale instead of the revised COVID-19 Threat Scale. The analyses described are identical to those reported in the publication.

***Results***

As expected, COVID-19 threat levels were associated with greater activation of the BIS, *r*(356) = .57, *p* < .001, 95% CIs [0.49, 0.66]. COVID-19 threat levels were also positively associated with ingroup entitativity, *r*(356) = .13, *p* = .012, 95% CIs [0.03, 0.24], control restoration motivation, *r*(356) = .13, *p* = .018, 95% CIs [0.02, 0.23], ingroup bias, *r*(356) = .11, *p* = .04, 95% CIs [0.01, 0.21] and marginally positively associated with passive party support, *r*(356) = .09, *p* = .077, 95% CIs [-0.01, 0.20]. No significant correlation emerged between COVID-19 threat levels and outgroup derogation *r*(356) = -.02, *p* = .646, 95% CIs [-0.13, 0.08].

To test the hypothesized indirect associations between COVID-19 threat levels and the defense strategies over behavioral inhibition, we ran simple mediation analyses via Hayes’ SPSS PROCESS macro (Version 3.4) separately for each of the defense variables that were found to correlate positively with the activation level of the BIS. This was the case for ingroup entitativity, *r*(356) = .18, *p* = .001, 95% CIs [0.08, 0.28], control restoration motivation, *r*(356) = .20, *p* < .001, 95% CIs [0.09, 0.30], and passive party support, *r*(356) = .24, *p* < .001, 95% CIs [0.14, 0.34]. Behavioral inhibition did not correlate significantly with ingroup bias, *r*(356) = .04, *p* = .411, 95% CIs [-0.06, 0.15], nor with outgroup derogation, *r*(356) = .03, *p* = .534, 95% CIs [-0.07, 0.14].

For each mediation analysis, 5000 bootstrap samples were created to establish a ninety-five percent bias corrected confidence interval for the expected indirect associations.

**Main Analyses.** Mediation analyses showed that the expected indirect effects of COVID-19 threat on distal defense strategies through BIS were significant for ingroup entitativity, control restoration motivation, and passive party support. Indirect effects were not significant for ingroup bias, *b* = -0.04, *SE* = 0.1, 95% CIs [-0.23, 0.15] and outgroup derogation, *b* = 0.12, *SE* = 0.12, 95% CIs [-0.11, 0.35]. Detailed statistical values for the significant indirect effects are presented for each DV separately in the following.

***Ingroup Entitativity.*** The regression coefficient between COVID-19 threat levels and ingroup entitativity was significant (total effect: *b* = 0.35, *SE* = 0.14, *t*(356) = 2.53, *p* = .012, 95% CIs [0.08, 0.61]). Importantly and as predicted, the indirect effect of COVID-19 threat levels via behavioral inhibition levels on ingroup entitativity was significant, *b* = 0.23, *SE* = 0.1, 95% CIs [0.05, 0.42]. Furthermore, the regression coefficient between behavioral inhibition levels and ingroup entitativity when controlling for the variance shared by COVID-19 threat levels and ingroup entitativity was significant, *b* = 0.25, *SE* = 0.1, *t*(356) = 2.47, *p* = .014, 95% CIs [0.05, 0.44]. The direct effect of COVID-19 threat levels on ingroup entitativity when controlling for the variance shared by behavioral inhibition level and ingroup entitativity, was non-significant, *b* = 0.11, *SE* = 0.17, *t*(356) = 0.68, *p* = .50, 95% CIs [-0.21, 0.44].

***Control Restoration Motivation.*** The total effect of COVID-19 threat levels on control restoration motivation was significant, *b* = 0.43, *SE* = 0.18, *t*(356) = 2.38, *p* = .018, 95% CIs [0.08, 0.79]. Importantly and as predicted, the indirect effect of COVID-19 threat levels via behavioral inhibition levels on control restoration motivation was significant, *b* = 0.36, 95% CIs [0.11, 0.64]. Furthermore, the regression coefficient between behavioral inhibition levels and control restoration motivation when controlling for the variance shared by COVID-19 threat levels and control restoration motivation was significant, *b* = 0.38, *SE* = 0.13, *t*(356) = 2.90, *p* = .004, 95% CIs [0.12, 0.65]. The direct effect of COVID-19 threat levels on control restoration motivation was non-significant, *b* = 0.07, *SE* = 0.22, *t*(356) = 0.31, *p* = 0.754, 95% CIs [-0.36, 0.5].

***Passive Party Support.*** The regression coefficient between COVID-19 threat levels and passive party support (i.e., total effect) was marginally significant, *b* = 0.31, *SE* = 0.17, *t*(356) = 1.77, *p* = .077, 95% CIs [-0.04, 0.66]. Importantly and as predicted, the indirect effect of COVID-19 threat levels via behavioral inhibition levels on passive party support was significant, *b* = 0.54, *SE* = 0.14, 95% CIs [0.27, 0.82]. The regression coefficient between behavioral inhibition levels and passive party support when controlling for the variance shared by COVID-19 threat levels and passive party support was significant, *b* = 0.57, *SE* = 0.13, *t*(356) = 4.45, *p* < .001, 95% CIs [0.32, 0.82]. The direct effect of COVID-19 threat levels on passive party support was non-significant, *b* = -0.22, *SE* = 0.21, *t*(356) = 1.05, *p* = .295, 95% CIs [-0.64, 0.19].

**Exploratory Analyses.** In order to better understand the non-significant correlation between activation levels of the behavioral inhibition system and ingroup bias, we ran exploratory mediation analyses for the two components of ingroup bias, namely outgroup warmth/competence rating and ingroup warmth/competence rating.

***Outgroup Warmth/Competence.*** The regression coefficient between COVID-19 threat levels and outgroup warmth/competence rating (i.e., total effect) was non-significant, *b* = - 0.1, *SE* = 0.12, *t*(356) = 0.88, *p* = .382, 95% CIs [-0.34, 0.13]. The indirect effect of COVID-19 threat levels via behavioral inhibition levels on outgroup warmth/competence rating was significant, *b* = 0.18, *SE* = 0.09, 95% CIs [0.02, 0.36].

Furthermore, the regression coefficient between behavioral inhibition levels and outgroup warmth/competence rating when controlling for the variance shared by COVID-19 threat levels and outgroup warmth/competence was significant, *b* = 0.19, *SE* = 0.09, *t*(356) = 2.21, *p* = .028, 95% CIs [0.02, 0.36]. The direct effect, namely the regression coefficient between COVID-19 threat levels and outgroup warmth/competence rating was significant, *b* = -0.29, *SE* = 0.14, *t*(356) = 1.99, *p* = .048, 95% CIs [-0.57, -0.003].

***Ingroup Warmth/Competence.*** The regression coefficient between COVID-19 threat levels and ingroup warmth/competence rating (i.e., total effect) was significant, *b* = 0.18, *SE* = 0.09, *t*(356) = 2.00, *p* = .046, 95% CIs [0.003, 0.36]. The indirect effect of COVID-19 threat levels via behavioral inhibition levels on ingroup warmth/competence rating was significant, *b* = 0.14, *SE* = 0.07, 95% CIs [0.005, 0.28]. Furthermore, the regression coefficient between behavioral inhibition levels and ingroup warmth/competence rating when controlling for the variance shared by COVID-19 threat levels and ingroup bias was significant, *b* = 0.15, *SE* = 0.07, *t*(356) = 2.23, *p* = .026, 95% CIs [0.02, 0.28]. The direct effect, namely the regression coefficient between COVID-19 threat levels and ingroup warmth/competence rating was non-significant, *b* = 0.04, *SE* = 0.11, *t*(356) = 0.374, *p* = .709, 95% CIs [-0.18, 0.26].
